# *In vitro* evaluation of the automated hematology analyzer XN-31 for rapid diagnosis of equine piroplasmosis

**DOI:** 10.1128/spectrum.00582-24

**Published:** 2024-09-13

**Authors:** Akihiro Ochi, Yuji Toya, Mikako Sengoku, Seiichiro Tsuchiya, Daiki Kishi, Takanori Ueno

**Affiliations:** 1 Equine Research Institute, Japan Racing Association, Shimotsuke, Tochigi, Japan; 2 Cell Technology, Engineering 1, Sysmex Corporation, Kobe, Hyogo, Japan; Central Texas Veterans Health Care System, Temple, Texas, USA

**Keywords:** equine piroplasmosis, rapid diagnosis, hematology analyzer, XN-31, horse

## Abstract

Equine piroplasmosis (EP) is a protozoal disease affecting equids, caused by *Theileria equi* and *Babesia caballi*. EP is conventionally diagnosed using microscopic, molecular, and/or serological methods, which are time-consuming. Consequently, there is a need for faster testing methods. In this study, we evaluated the application of the Sysmex XN-31 automated hematology analyzer, originally a rapid test for detecting malaria in humans, for the diagnosis of EP. The cultured parasites were measured using the XN-31 that had been customized for horse blood samples (XN-31m). The following parameters were evaluated: limit of detection (LoD), limit of quantification (LoQ), linearity, carryover, precision, and correlation with microscopic examination. The XN-31m detected infected red blood cells (RBCs) in approximately 1 minute. The LoD and LoQ for *B. caballi* were 4.54 infected RBCs/μL and 14.10 infected RBCs/μL, while those for *T. equi* were 5.80 infected RBCs/μL and 11.44 infected RBCs/μL, respectively. Linearity showed excellent correlation (*R*^2^ > 0.99), and carryover never exceeded 0.5%. The coefficient of variation was under 5%. The correlation between the results obtained using XN-31m and microscopic examination was high (*R*^2^ > 0.98). In conclusion, the XN-31 analyzer detected *B. caballi* and *T. equi* parasites in approximately 1 minute with high sensitivity. The results indicate the potential of the XN-31 analyzer as a fast and user-friendly diagnostic method for EP.

**IMPORTANCE:**

In this study, we demonstrated that the automated hematology analyzer, XN-31, can detect red blood cells infected with *Babesia caballi* and *Theileria equi* in about 1 minute. We evaluated the diagnostic performance of the XN-31 analyzer for equine piroplasmosis, providing evidence of its potential as a diagnostic tool for this disease.

## INTRODUCTION

Equine piroplasmosis (EP) is a tick-borne disease affecting equines (horses, donkeys, mules, and zebras) and is caused by the intra-erythrocytic protozoa *Babesia caballi* and *Theileria equi* (1). Clinical signs of EP include fever, hemolytic anemia, red urine, jaundice, edema, and potentially death (2, 3). However, these clinical signs are often non-specific, and most infected horses show no clinical signs, becoming lifelong carriers and serving as reservoirs for transmission to naïve horses in endemic regions (1). These parasites are widely distributed throughout the world, including in Europe, Asia, Africa, and South America, with approximately 90% of equids worldwide living in areas endemic for EP (4). Due to the socioeconomic impact of the disease, EP is listed as one of the notifiable diseases of equids by the Terrestrial Animal Health Code of the World Organization for Animal Health (WOAH) (5). Many countries also require EP inspections for international and, sometimes, intra-country transfers of horses (6).

Laboratory diagnostic methods for EP rely on the identification of parasites and specific antibodies (7). Common serological tests include the indirect immunofluorescence assay (IFAT), complement fixation test, and competitive enzyme-linked immunosorbent assay (ELISA), which are used to identify chronically infected horses. Specifically, IFAT and cELISA are the primary tests used for qualifying horses for importation (7). While IFAT is sensitive, it is time-consuming and highly subjective, particularly in interpreting fluorescence, and requires a large quantity of antigens. Conversely, cELISA, recommended by the United States Department of Agriculture (USDA) and WOAH for international horse transport, uses purified recombinant antigens, RAP1 and EMA1/EMA2, for *B. caballi* and *T. equi*, respectively. However, challenges arise due to the genetic variability of *rap-1* gene in *B. caballi* isolates leading to detection failures in some cases with USDA-approved cELISA (8, 9). Also, recent studies have reported that false negative results might be associated with cELISA for *T. equi* due to the absence of the *EMA1* gene in *Theileria haneyi*, formerly known as *T. equi* clade C (10). Moreover, the necessity for separate tests for *B. caballi* and *T. equi* makes serological tests more laborious. Furthermore, early-stage infections may yield false-negative results until the antibodies reach levels detectable by these serodiagnostic methods (11, 12).

For an accurate diagnosis of EP, microscopic examination of blood smears and/or PCR in combination with serological techniques is recommended in the WOAH manual (7). Microscopic examination of Giemsa-stained thin blood smears, being simple and useful for demonstrating parasites in red blood cells (RBCs), is still used today in most diagnostic laboratories and clinical settings. However, this method is labor intensive and has low sensitivity, and its success is dependent on the experience and expertise of the examiner. It particularly struggles with the detection of low parasitemia levels in subclinical and chronic infections. In contrast, molecular diagnostic methods, such as PCR (13, 14), nested PCR (15, 16), and real-time PCR (17, 18), are more sensitive but expensive and require skilled laboratory techniques. In addition, these tests take 3–6 hours to yield results, limiting their utility for large-scale use, such as screening assays. Therefore, there is a need for a sensitive, user-friendly, and field-deployable diagnostic test for EP.

An innovative assay has recently been developed to meet these requirements, using the automated hematology analyzer XN-31 (Sysmex Corp., Kobe, Japan). This CE-marked platform was developed to support the diagnosis of human malaria in clinical settings. The XN-31 analyzer has the same physical characteristics as the XN-30 developed for research use. It operates on the principle of fluorescence flow cytometry, which counts and classifies cells by irradiating them with a 405 nm laser beam, analyzing the resultant forward-scattered light (FSC) to reflect the size of infected RBCs and side fluorescent light (SFL) indicating nucleic acid content (19, 20). The XN-31 analyzer is also able to distinguish between the malarial species *Plasmodium falciparum* and *Plasmodium vivax* (19) and detect their gametocytes (20, 21) with good sensitivity and specificity. A previous study has reported that the limit of detection (LoD) is 5.9 infected cells/µL (22). The parasitemia levels determined by the XN-31 analyzer show a strong correlation with those obtained from microscopic analysis of cultured malaria and mouse/human blood samples (23–25). This innovative, user-friendly, and rapid testing method, using a blood cell counter, is expected to serve as an alternative to PCR and microscopy in diagnosing human malaria.

Due to these attributes, the XN-31 analyzer should be potentially capable of detecting RBCs infected with *B. caballi* and *T. equi*. The objective of this study was to evaluate the performance of the XN-31 analyzer in the rapid diagnosis of EP.

## MATERIAL AND METHODS

### Parasite culture

*B. caballi* (USDA strain) and *T. equi* (USDA strain) were cultured *in vitro* in an atmosphere of 5% O_2_ and 5% CO_2_ at 37°C, as previously described (26). The culture medium for *B. caballi* and *T. equi* was RPMI1640 medium (Sigma-Aldrich, Tokyo, Japan) and Medium 199 (Sigma-Aldrich), respectively, each supplemented with 40% horse serum, 13.6 µg/mL of hypoxanthine, 1% GlutaMAX-I (all from Sigma-Aldrich), and horse RBCs at a 10% hematocrit. The horse serum and RBCs were collected from healthy horses and prepared as previously described (27). The collected whole blood was defibrinated immediately using glass beads and centrifuged at 500 × *g* for 20 minutes at 4°C. The serum was decanted and stored at −80°C until use. The buffy coat was discarded, and the remaining erythrocytes were suspended in the appropriate culture medium and stored at 4°C until future use.

This study was approved by the Institutional Animal Care and Use Committee (Permission number: 21–12) and carried out according to the Equine Research Institute Animal Experimentation Regulations.

### Measurement by automated hematology analyzer XN-31 and data analysis

*In vitro* cultured *B. caballi*, *T. equi*, and their mixed samples were analyzed using the XN-31 analyzer (Sysmex Corp., Kobe, Japan) modified for horse blood testing (XN-31m) and set to low malaria (LM) mode . For the measurements, dedicated reagents (CELLPACK DCL, SULFOLYSER, Lysercell M, and Fluorocell M; Sysmex Corp.) were used, according to the manufacturer’s instructions. The RBC count (RBC#) was determined using the sheath flow direct current detection method, and the corresponding count of infected RBCs (MI-RBC#; reported as infected RBC per microliter) was measured using the XN-31 fluorescence flow cytometry-based technique.

For manual analysis, flow cytometry standard data exported from the XN-31m analyzer were analyzed using FlowJo version 10.8.1 (BD Biosciences, Ashland, OR, USA). Areas of infected RBCs and non-infected RBCs were designated on the scattergram (SFL-FSC), and the dots representing infected RBCs in the gated area were counted. The scattergram presented dots corresponding to 0.953 µL of sample volume, whereas the analyzer reported total RBC counts per 1 µL. This difference was compensated in the calculation of parasitemia, as shown in the following equation:


Parasitemia (%)=infected RBC# (counts)/ 0.953/ 3/ total RBC# (counts/μL)×100

### Precision (within-run repeatability)

To evaluate the precision of the XN-31m analyzer, samples with varying levels of parasitemia for *B. caballi* and *T. equi* were tested. These samples were categorized into four groups based on their parasitemia levels: high (>1.5%), medium (1.0%–1.5%), low (0.5%–1.0%), and very low (<0.5%). Each group was tested 10 consecutive times. For the total RBC counts, infected RBC counts, and parasitemia, the mean, SD, and coefficient of variation (CV%) were calculated.

### Linearity

Linearity in diagnostic testing is the ability to provide results that are directly proportional to the concentration of the analyte being measured over a specific range. The linearity of the XN-31m analyzer was evaluated by analyzing three repeats of a 10-point (0%, 10%, 20%, 30%, 40%, 50%, 60%, 70%, 80%, 90%, and 100%) serial dilution of known parasitemia samples with a diluent solution (CELLPACK DCL) (28).

### Carryover

Carryover was evaluated according to the CLSI H26-A2 guideline, which covers the assessment of high parasitemia (H1, H2, and H3) and low parasitemia (L1, L2, and L3) samples (*B. caballi* and *T. equi*, respectively) in a sequence of three consecutive tests (28). First, the high parasitemia samples were analyzed, followed by the low parasitemia samples, to observe the influence of preceding high parasitemia measurements on subsequent low parasitemia readings. We assessed the percentage of carryover using the formula:


[(L1−L3)/(H3−L3)]×100

### Limit of blank, limit of detection, and limit of quantification

To determine the lower detection threshold of the XN-31m analyzer, we evaluated the limit of blank (LoB), LoD, and limit of quantification (LoQ). The LoB was estimated using blank samples (CELLPACK DCL) and determined as the 95th percentile of the blank samples’ distribution, calculated non-parametrically based on 70 values. The LoD was assessed by measuring seven standard samples with different parasitemia levels. Each standard sample was measured five consecutive times daily, over a 3-day period. The LoD was calculated as LoD = LoB + Cp × SD, where Cp is the coefficient corresponding to the 95th percentile of a normal distribution. The LoQ was determined based on the point where the CV of measurements from the same dilution series did not exceed 20%.

### Comparison of microscopic examinations

For microscopic examination, thin blood smears were prepared, stained with May-Grünwald reagent (Muto Pure Chemicals, Tokyo, Japan) at room temperature for 5 minutes, incubated with PBS (pH 6.4) for 10 minutes, and then incubated with 1:20 diluted Giemsa solution (Muto Pure Chemicals) at room temperature for 20 minutes. The stained slides were examined under a model BX53 light microscope (Olympus, Tokyo, Japan) at 1,000× magnification. The percentage of infected RBCs (parasitemia) was determined by counting infected RBCs in at least 10,000 erythrocytes by an experienced pathologist.

### Statistical analyses

Statistical analyses were conducted to assess the reliability and precision of the data collected in this study. Key statistical metrics calculated included the coefficient of determination (*R*^2^), mean, SD, and coefficient of variation (CV% = SD/mean × 100). These calculations were performed using Excel software for Mac 2019 (Microsoft, Redmond, WA, USA).

## RESULTS

### Detection of non-infected and infected RBCs

**Fig 1:**
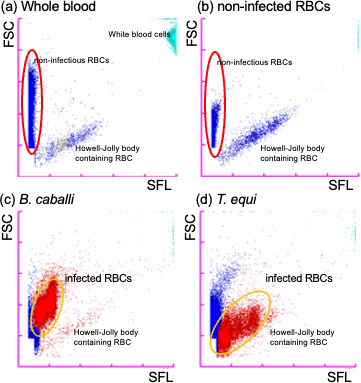
XN-31 analyzer data of blood samples from non-infected and infected samples. M scattergrams were obtained from a healthy horse whole blood sample (a), non-infected RBCs (b), *B. caballi*-infected RBCs (c), and *T. equi-*infected RBCs (d). Blue, non-infected RBCs; light blue, white blood cells; and red, infected RBCs were assigned based on the default setting of the XN-31 analyzer. *B. caballi-* and *T. equi*-infected RBCs were depicted as distinct clusters, forming two or three clusters, respectively. Howell-Jolly bodies containing RBCs were occasionally misclassified as infected RBCs in the equine blood samples.

To apply the XN-31m analyzer for the diagnosis of EP, we first analyzed peripheral blood samples from healthy horses. A malaria (M) scattergram is generated for each sample and consists of SFL intensity (corresponding to the amount of nucleic acid) on the horizontal axis (x-axis) and FSC intensity (indicating the size of cells) on the vertical axis (y-axis; Fig. 1). The XN-31m analyzer detected white blood cells, non-infected RBCs, and RBCs containing Howell-Jolly bodies (HJB-RBCs) on the M scattergram (Fig. 1). Non-infected RBCs were formed a cluster on the left side of the M scattergram, along the vertical axis. White blood cells are larger in size and have a higher nucleic acid content, forming a cluster in the top right-hand corner of the M scattergram.

In the evaluation of cultured samples infected with *B. caballi* and *T. equi*, the XN-31m analyzer distinguished infected RBCs (red dots) from non-infected RBCs (blue dots) on the M scattergram in approximately 1 minute. Moreover, the XN-31m distinguished *B. caballi*-infected RBCs from *T. equi*-infected RBCs, representing them on the M scattergrams as separate clusters (Fig. 1; Fig. S1). HJB-RBCs were often misidentified as EP-infected RBCs, as in a previous study on rodents (23), although they formed a distinct cluster from the infected RBCs (Fig. 1). The low prevalence of HJB-RBCs did not affect the detection of infected RBCs using the XN-31m. However, these findings suggest a limitation in accurately counting infected RBCs by the XN-31m due to the presence of HJB-RBCs, indicating a need to re-analyze the data to calculate the number of infected RBCs. We used manual gating to count infected and uninfected RBCs on the scattergrams, as described in the Materials and methods.

### Precision (within-run repeatability)

The precision of infected RBC counts, total RBC counts, and parasitemia measurements for samples infected with *B. caballi* and *T. equi* is shown in Table 1. For samples infected with *B. caballi* and *T. equi*, the CV%s of infected RBC counts ranged from 0.82% to 2.26% and 0.61% to 1.72%, respectively. The CV%s of other parameters were also acceptable (<2.5%).

**TABLE 1:** Precision (within-run repeatability) of the XN-31m^*a*^

		*B. caballi*			*T. equi*		
Parasitemia		Total RBC	Infected RBC	Parasitemia	Total RBC	Infected RBC	Parasitemia
		(cells/µL)	(cells/µL)	(%)	(cells/L)	(cells/µL)	(%)
High	Mean	947,000	18393.98	1.94	906,000	23682.76	2.61
(>1.5%)	SD	2603.42	47.63	0.01	1632.99	45.86	0.01
	CV%	0.87	0.82	0.95	0.57	0.61	1.06
Middle	Mean	938,000	11062.82	1.18	982,000	12682.76	1.29
(1.0%–1.5%)	SD	4898.98	39.96	0.01	3590.11	22.06	0.00
	CV%	1.65	1.14	1.40	1.16	0.55	1.11
Low	Mean	1,021,000	5463.55	0.54	1,033,000	6139.07	0.59
(0.5%–1.0%)	SD	3480.1	27.97	0.00	4484.54	16.52	0.00
	CV%	1.08	1.62	1.49	1.37	0.85	1.56
Very low	Mean	997,000	849.18	0.09	1,067,000	970.90	0.09
(0.5%>)	SD	4229.53	6.06	0.00	5385.16	5.29	0.00
	CV%	1.34	2.26	1.93	1.60	1.72	2.14

^*a*^ Data are mean, SD, and coefficient of variation (CV) for infected samples with very low, low, middle, and high parasitemia.

### Linearity

**Fig 2:**
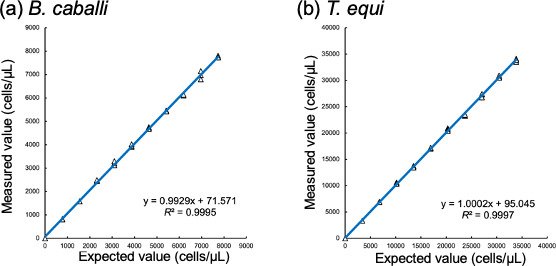
Linearity of the XN-31m analyzer. (**a**) *B. caballi*-infected sample. (**b**) *T. equi-*infected samples (**b**). X-axis: the expected value of infected RBCs; Y-axis: measured value by the XN-31 analyzer. *R*^2^ indicates the coefficient of determination.

The linearity was evaluated by serially diluting *in vitro* cultured samples infected with *B. caballi* and *T. equi*. Using this approach, the correlation coefficients were excellent (*R*^2^ > 0.999) between expected theoretical concentrations and obtained values of parasitemia for samples infected with *B. caballi* and *T. equi* (Fig. 2).

### Carryover

Carryover is defined as the amount of analyte carried by the analyzer from one sample measurement into the subsequent measurement. The results showed that carryover never exceeded 0.2% (Table 2).

**TABLE 2:** Carryover of the XN-31m

Parasites	Carryover (%)
*B. caballi*	0.198
*T. equi*	0.144

### Limit of blank, limit of detection, and limit of quantification

The detection limits of the XN-31m analyzer for EP are summarized in Table 3. The LoB was 0.70 infected RBCs/µL. A dilution series of *in vitro* cultured parasites was used to determine the LoD and LoQ. The LoD was 4.54 infected RBCs/µL for *B. caballi* and 5.80 infected RBCs/µL for *T. equi*. The LoQ was 14.10 infected RBCs/µL for *B. caballi* and 11.44 infected RBCs/µL for *T. equi* (Fig. S2).

**TABLE 3:** LoB, LoD, and LoQ of the XN-31m

Parasites	LoB	LoD	LoQ
	(Infected RBCs/µL)	(Infected RBCs/µL)	(Infected RBCs/µL)
*B. caballi*	0.70	4.54	14.10
*T. equi*	0.70	5.80	11.44

### Comparison between the XN-31 analyzer and microscopic examination

**Fig 3:**
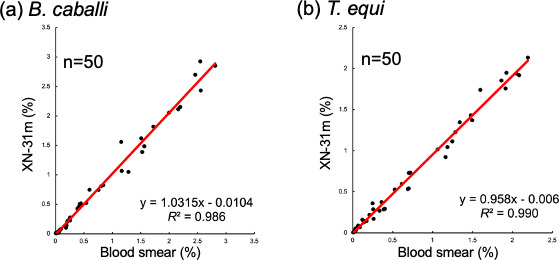
Correlation of parasitemia between the XN-31m and microscopic examination. Samples were analyzed in LM mode. *R*^2^ indicates the coefficient of determination. The diagonal line represents the regression line.

To investigate the reliability of the XN-31m, its ability to measure parasitemia was compared with traditional microscopic examination of Giemsa-stained smears. The correlation analysis showed a strong coefficient of determination for *B. caballi* (*R*^2^ = 0.986) and *T. equi* (*R*^2^ = 0.990; Fig. 3).

## DISCUSSION

Increased international transport of horses has created a need for more accurate and rapid EP testing methods. The purpose of EP testing is usually to declare an animal free from infection, to confirm suspicion of infection, or to confirm the efficacy of treatment. EP diagnosis largely relies on serological tests, blood smears, and molecular methods; however, despite their effectiveness, these methods are time-consuming and require highly trained examiners and laboratory equipment for accurate testing (1). The increasing demand for highly accurate and rapid EP testing has created an unmet need. In this study, we demonstrated that the XN-31m analyzer can detect RBCs infected with *B. caballi* and *T. equi* in a rapid and automated manner with high sensitivity.

Given the excellent performance and practicability of the XN-31m, this automated analyzer emerges as a promising novel diagnostic method for EP. Rapid and accurate diagnosis of EP is essential for successful control and prevention of the disease. The WOAH recommends a combined approach of serological tests and PCR/blood smear for the diagnosis of EP (7). Serological methods, such as IFAT and cELISA, are widely used to identify chronic cases. However, during the early stage of the infection, horses may not have detectable levels of antibodies for serological tests to be positive, although they might still test positive by blood smears and PCR. The diagnosis of EP involves serodiagnosis and agent identification tests, which are time-consuming. Our data suggest that the XN-31 would be a game changer as a novel diagnostic method for EP.

The LoD of the XN-31m is less than 10 infected RBC/µL, suggesting its high sensitivity as a method for diagnosing EP. Microscopic examination of blood smears has been the most conventional method for confirming *Babesia* spp. and *Theileria* spp. infections in animals (1, 27, 29) and malaria in humans (30, 31). The sensitivity of blood smear analysis for detecting babesiosis ranges from 10^−5^% to 10^−6^% parasitemia (single-infected RBC per 10^5^–10^6^ RBCs) (32). In the case of human malaria, the LoD of a blood smear is at about 1–2 × 10^−3^% parasitemia (50–100 infected RBCs/μL, assuming an average total RBC count of 5 × 10^6^ cells/µL of blood in humans) under optimum conditions by experienced examiners (31). Molecular methods for diagnosing EP are more sensitive than microscopic examinations. The highest analytical sensitivity values have been reported for nested PCR and qPCR methods. Specifically, nested PCR can detect fewer than 10 parasites of *T. equi* (33) and a single infected cell of *B. caballi* (34). Moreover, real-time PCR assays for *T. equi* have demonstrated the capability to detect as few as 2.5 infected RBCs/μL (at least 2–3.8 × 10^−5^% parasitemia) (35). The parasitemia during clinical infections caused by *B. caballi* does not exceed 1% and may be less than 0.1% (1,000 infected RBCs/µL assuming a total RBC count of 10^6^ cells/µL of blood), while during clinical diseases caused by *T. equi*, the parasitemia is usually between 1% and 5% (1–5 × 10^4^ infected RBCs/µL) (1). Taken together, the XN-31 analyzer is considered sensitive enough to detect such acute cases of EP. However, in chronic or subclinical EP cases, the parasitemia is usually too low for reliable detection by blood smears. Although this study showed that the XN-31m analyzer has high sensitivity, further *in vivo* evaluations are required to determine whether it can detect chronic cases of EP.

To evaluate the instrument’s ability to detect RBCs infected with *T. equi* and *B. caballi*, we first validated fluorescence and light-scattering parameters between samples infected with the parasites. The fluorescence and light scattering properties of *T. equi* and *B. caballi* were distinct on the M scattergrams, allowing for species identification (Fig. 1; Fig. S1). Within erythrocytes, *B. caballi* merozoites are pear-shaped, 2–5 µm in length and 1.3–3.0 µm in diameter, and sometimes occur in pairs. In contrast, *T. equi* merozoites in RBCs are relatively smaller, less than 2–3 µm, and are round or oval in shape, forming one to four separate merozoites (1). Considering that the XN-31 analyzer distinguishes the *Plasmodium* spp. by measuring DNA amount and internal cell structures (20, 21), the differences in parasite size, genome size (12.8 Mbp for *B. caballi* and 11.6 Mbp for *T. equi*) (36, 37), and number of parasites in RBCs may be responsible for the differences observed in the M scattergrams.

The default algorithm of the XN-31m analyzer was insufficient to accurately calculate the parasitemia of *B. caballi* and *T. equi*. This can be attributed to the tendency of the analyzer to misidentify HJB-RBCs as infected RBCs, as previously reported in mouse blood samples (38). HJBs are nuclear remnants and can occasionally be observed in healthy horses. These findings suggest that HJB-RBCs prevent accurate quantification of infected RBCs using the algorithm equipped in the XN-31m analyzer. This algorithm automatically gates a specific region of the M-scattergram, known as the M-gating area, and detects and counts RBCs within this region as malaria-infected RBCs. In this study, for accurate calculation of parasitemia, the infected RBC regions on M scattergrams were manually gated for every sample. Therefore, consideration regarding an automated gating method will be required to improve the performance of diagnosis for EP. It is recommended that other testing methods should be used in combination with the XN-31m when a suspicious scattergram is obtained.

The XN-31 analyzer detects malaria parasites using flow cytometry technology (21, 25, 39). This technology has been used to detect malaria and babesia parasites since the 1970s, as it provides a more accurate and low-labor alternative to manual cell counting through microscopy. Most flow cytometry-based methods use DNA/RNA staining dyes, such as SYBR green and SYTO16, to distinguish infected RBCs from their uninfected counterparts (40, 41). Although flow cytometry has high sensitivity, its implementation in the field would require well-trained technicians, and the methods can be time-consuming due to the processing of specimens and the setup required for detection, in addition to expensive equipment. Consequently, flow cytometry-based methods are not yet practical for diagnosing babesiosis/theileriosis in animals. In contrast, the XN-31 analyzer aspirates approximately 60 µL of blood directly from a blood collection tube and analyzes it automatically. The entire testing process of the XN-31m analyzer takes approximately 1 minute. No sample processing is required, allowing for high-throughput testing of targeted horses. These performances are supported by a combination of two dedicated reagents, Lysercell M (a lysate containing a non-ionic surfactant) and Fluorocell M (a solution containing the nucleic acid stain Hoechst dye) with a blue laser.

Owing to its property of directly detecting parasites in RBCs, the XN-31 analyzer has the potential to detect blood protozoa other than *T. equi* and *B. caballi. T. haneyi*, a newly identified piroplasm affecting equids, has recently been reported in many countries (10), complicating the diagnostic workflow for EP. The XN-31 analyzer might simultaneously detect *T. haneyi,* other intra-erythrocytic parasites, and Trypanosoma. Another important advantage of the XN-31 analyzer is that it also provides a conventional complete blood count with each analysis, which is not offered by other diagnostic tools for EP. This provides clinical veterinarians important information regarding the hematological status of the animal during infection and treatment, in addition to the parasitemia.

In conclusion, the XN-31m analyzer holds promise as a rapid and sensitive diagnostic method for EP. It can be used to detect horses with low-density parasitemia and is a useful screening tool in EP control and prevention programs. Deployment of this device at strategic locations could lead to improved disease management, treatment, and screening, ultimately contributing to more effective containment and mitigation of EP.

## SUPPLEMENTAL MATERIAL

Figure S1 spectrum.00582-24-s0001.tiff XN-31 analyzer data of blood samples from mixed infected sample.

Figure S2 spectrum.00582-24-s0002.tiff Limit of quantification (LoQ) of the XN-31m.

ASM does not own the copyrights to Supplemental Material that may be linked to, or accessed through, an article. The authors have granted ASM a non-exclusive, world-wide license to publish the Supplemental Material files. Please contact the corresponding author directly for reuse.
