# Supplementary figures and images for: In vitro evaluation of the automated hematology analyzer XN-31 for rapid diagnosis of equine piroplasmosis
Source: Microbiol Spectr. 2024 Sep 13;12(10):e00582-24. doi: 10.1128/spectrum.00582-24 (PMC11448420; doi:10.1128/spectrum.00582-24)

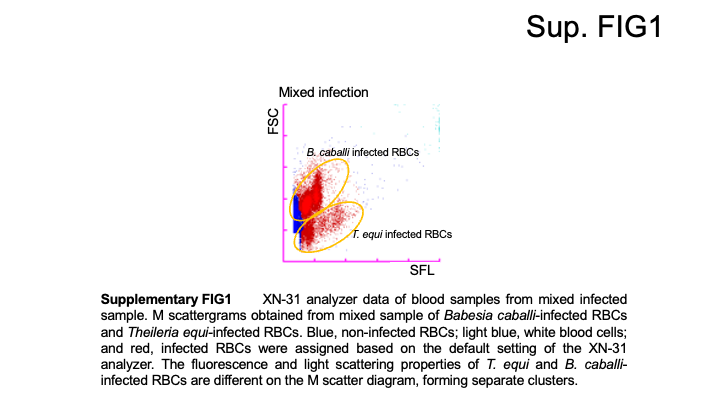

Supplement: Figure S1 — XN-31 analyzer data of blood samples from mixed infected sample. [file spectrum.00582-24-s0001.tiff]

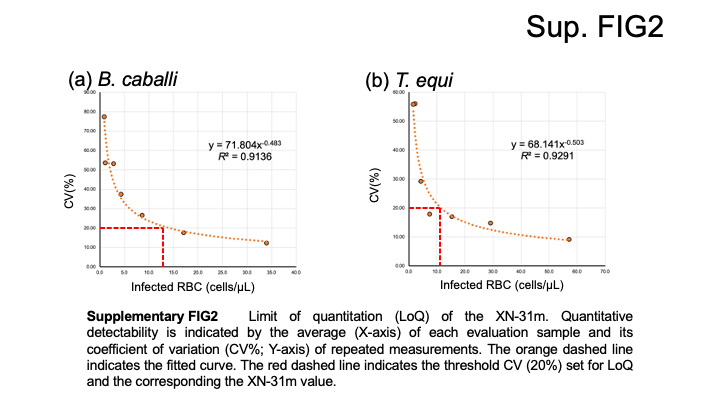

Supplement: Figure S2 — Limit of quantification (LoQ) of the XN-31m. [file spectrum.00582-24-s0002.tiff]
